# The Benign Impostor: Recognizing a Multinodular and Vacuolating Neuronal Tumor to Prevent Unnecessary Intervention

**DOI:** 10.7759/cureus.95557

**Published:** 2025-10-28

**Authors:** Sabrina Amaouche, Amin Da'meh, Mohammad G Deameh, Pir Abdul Ahad Aziz Qureshi, Vikram Rao Bollineni

**Affiliations:** 1 Department of Radiology, Universitair Ziekenhuis Brussel, Brussels, BEL; 2 Clinical Research, Al-Balqa Applied University, Salt, JOR; 3 Faculty of Medicine, Háskóli Íslands, Reykjavik, ISL; 4 Department of Radiology, Landspítali - The National University Hospital of Iceland, Reykjavik, ISL

**Keywords:** adult-onset epilepsy, benign brain tumor, epileptogenic lesions, multinodular vacuolating neuronal tumor, seizure

## Abstract

A multinodular and vacuolating neuronal tumor (MVNT) is a rare, benign lesion often linked to adult-onset seizures. We report the case of a 36-year-old woman presenting with focal and generalized seizures, preceded by visual aura. MRI revealed classical MVNT features in the left parietal lobe without mass effect, enhancement, or diffusion restriction. Follow-up at 12 months demonstrated lesion stability. Seizure control was achieved with levetiracetam. Recognizing MVNT’s distinct radiological features is critical for avoiding unnecessary biopsy or surgical intervention. Conservative management with antiepileptic therapy and scheduled MRI surveillance should be followed in clinically stable cases.

## Introduction

A multinodular and vacuolating neuronal tumor (MVNT) is a rare, benign neoplasm of the central nervous system, particularly noted as a potential etiology for seizures that manifest in adults. First characterized by Huse et al. in 2013 and subsequently classified as a WHO Grade I tumor in 2016, MVNT falls within the broader category of glioneuronal tumors [1]. Despite the uncertainties surrounding its precise prevalence and underlying pathophysiological mechanisms, MVNT is considered a developmental malformation rather than a true neoplasm, with vacuolated neuronal cells and absence of mitotic activity histologically. Its prevalence is extremely low, with fewer than 100 cases reported in the literature. On MRI, MVNT typically appears as clusters of nodular lesions in the deep cortical ribbon and superficial subcortical white matter, exhibiting hyperintensity on T2-weighted and fluid-attenuated inversion recovery (FLAIR) sequences. These lesions demonstrate no enhancement after gadolinium, no diffusion restriction, and no surrounding edema or mass effect. They remain stable over time, earning the description “leave me alone” lesions [2]. Its estimated prevalence is less than 0.1% of all brain neoplasms, with fewer than 100 cases reported in the literature to date, underscoring its rarity and the importance of recognizing this entity.

The differential diagnosis of MVNT in adults may include enlarged perivascular spaces, dysembryoplastic neuroepithelial tumors (DNETs), low-grade gliomas (LGGs), and focal cortical dysplasia (FCD). In children and adolescents, ganglioglioma and DNET may mimic MVNT [3]. No biopsies for histopathologic confirmation are necessary in the majority of cases [2]. Early recognition is essential to prevent unnecessary intervention, as biopsy or surgical resection is rarely needed unless atypical features or symptomatic progression occur.

## Case presentation

A 36-year-old woman with no significant medical history presented with a two-year history of recurrent seizures. These included generalized tonic-clonic seizures, absence seizures, and focal motor seizures involving the right upper limb. A visual aura often preceded seizures. She also experienced intermittent diffuse headaches responding to over-the-counter pain medications, sometimes associated with seizures. These headaches had previously been considered migraine, and levetiracetam (Keppra) was prescribed solely for seizure management, not as antimigraine therapy.

**Figure 1:**
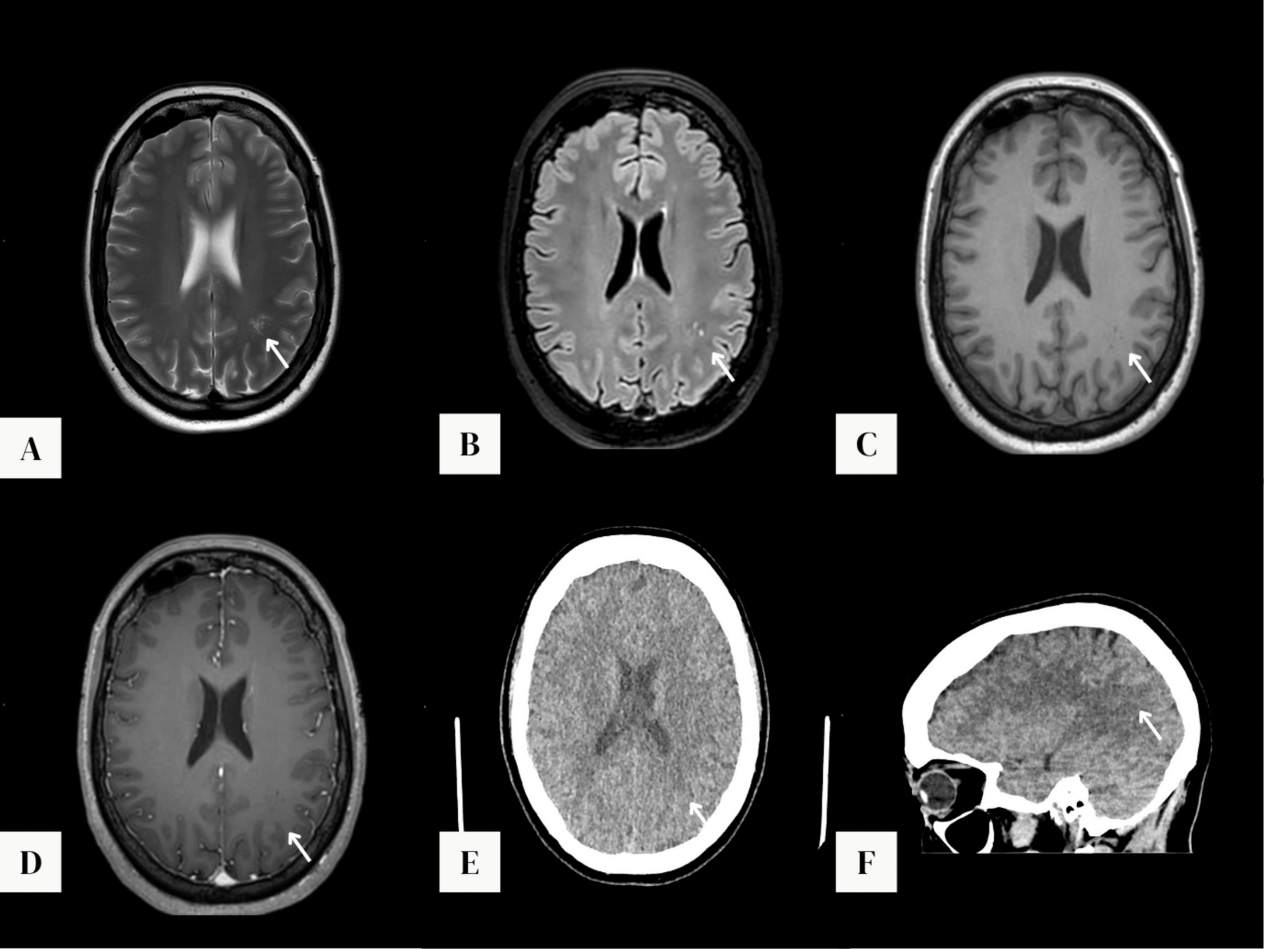
Imaging characteristics of a multinodular and vacuolating neuronal tumor in the left parietal lobe: MRI and CT features. Axial T2 image (A) and axial fluid-attenuated inversion recovery image (B) demonstrate a subcortical cluster of hyperintense nodules in the left parietal lobe (white arrows). Pre-contrast T1 (C) and post-contrast T1 (D) images show a cluster of slightly hypointense lesions in the grey matter without enhancement (white arrows). Brain CT in axial (E) and sagittal (F) planes reveals a hypodense area in the left parietal lobe with subcortical topography, with partially lobulated contour, such as a cluster of hypodense nodules (white arrows).

MRI revealed a cluster of sharply demarcated subcortical lesions in the left parietal lobe. These lesions were hyperintense on T2-weighted and FLAIR sequences, hypointense on T1-weighted images, showed no contrast enhancement after gadolinium administration, and showed no restricted diffusion on diffusion-weighted imaging or apparent diffusion coefficient imaging. There was no surrounding edema or mass effect. On retrospective review, the previously performed CT scan demonstrated a subcortical hypodensity in the left parietal lobe with a partially lobulated contour, such as a cluster of hypodense nodules, corresponding to the cluster of hyperintense subcortical nodules visualized on MRI (Figure 1).

Electroencephalography (EEG) studies were conducted intermittently between the time of diagnosis and the one-year follow-up. The EEGs performed were normal, except for mild left parietotemporal lateralization without epileptiform discharges. Subsequent EEGs performed around six and twelve months after diagnosis were normal.

Levetiracetam was started to control the patient’s seizures. Because the lesion exhibited benign features and the patient remained neurologically stable without a mass effect, the team opted for clinical follow-up instead of surgery. The patient remained stable on antiepileptic therapy with a significant reduction in seizure frequency. Imaging was performed sequentially over the course of approximately one year. A follow-up MRI performed 12 months later demonstrated radiological stability, with no new lesions or interval growth, confirming the benign nature of the process, and surgical intervention was not indicated.

## Discussion

MVNT represents an unusual benign neoplasm of the central nervous system that tends to remain unchanged over time, characterized and distinguished by specific radiological features. This condition predominantly arises during adulthood with a median age of 43 years, ranging from 21 to 71 years, often presenting with epileptic seizures [4,5]. Although epilepsy, headache, and other nonspecific symptoms have been described as symptoms associated with MVNT, most lesions described in the literature are asymptomatic. They are discovered incidentally during MRI studies performed for chronic neurologic symptoms.

Our patient, a 36-year-old woman, presented with a clinical and radiological feature that aligns with previously reported MVNT cases, marked by the occurrence of epileptic seizures and stable MRI findings over time. The diagnostic MRI findings observed in our case were characterized by clusters of sharply defined subcortical nodules that exhibited hyperintensity on T2-weighted and FLAIR sequences, hypointensity on T1-weighted images, and the absence of contrast enhancement and diffusion restriction, aligning with previously reported findings in the academic literature. Cerebral localization is more frequent in relation to the cerebellum, most often affecting the temporoparietal lobes and less frequent involvement of the frontal and occipital lobes [6]. These lesions are usually not detected on CT scans, but some studies have described them as non-enhancing hypoattenuating abnormalities of the subcortical white matter [6-8]. For example, Karataş et al. (2023) described similar imaging features in two patients, showing stable lesions on subsequent imaging assessments, thereby confirming the benign and non-progressive nature of MVNT [9]. Nunes et al. (2017) conducted a comprehensive review of 33 MVNT cases, with biopsy confirmation obtained in only four patients. With a mean follow-up period of 37 months, radiographic evaluation demonstrated the absence of progression, confirming the non-aggressive nature of the lesion. These findings support conservative management strategies that avoid unnecessary surgical intervention in asymptomatic patients, aligning with our approach [6]. Similarly, Biyikli et al. (2023) conducted a retrospective analysis of 11 patients, documenting stable lesion size and morphology on follow-up MRIs over an average of 14.8 months. Their investigation reported a spectrum of clinical presentations ranging from headaches to seizures and transient neurological deficits, demonstrating the clinical heterogeneity of MVNT. Seizures were less frequent in their cohort compared to our case, indicating variability in presentation [10]. Seizure management was effectively achieved with medical therapy, supporting a conservative, non-surgical approach. These observations are consistent with the current literature, which suggests that MVNT represents a developmental malformation rather than a true neoplasm, further supporting a conservative approach when no alarming signs are present.

In adults, the main differential diagnoses for MVNT include enlarged perivascular spaces, LGGs, and gangliocytomas. In pediatric and adolescent patients, the differentials broaden to include gangliogliomas and DNETs. The MRI characteristics of MVNT and its main differential diagnoses are summarized in Table 1.

**Table 1:** MRI features of MVNT and its main differential diagnoses. MVNT: multinodular and vacuolating neuronal tumor; FCD: focal cortical dysplasia; DNET: dysembryoplastic neuroepithelial tumor; LGG: low-grade glioma; FLAIR: fluid-attenuated inversion recovery; CSF: cerebrospinal fluid

Entity	Enhancement	Location	FLAIR signal	Growth/Follow-up	Key features
MVNT	None	Subcortical WM	Hyperintense	Stable	Clustered nodules
FCD	None	Cortical	Hyperintense	Stable	Gray-white blurring
DNET	Rare	Cortical	Mixed	Stable	Bubbly cortex
LGG	Rare/Patchy	Cortical-subcortical	Hyperintense	Progressive	Infiltrative margin
Perivascular spaces	None	Periventricular/Basal ganglia	Suppressed on FLAIR	Stable	CSF signal
Ganglioglioma	Often	Cortical	Hyperintense	Slow growth	Cystic-solid, calcified

There is a current debate regarding the optimal treatment of epilepsy associated with MVNT, between choosing antiepileptic drug treatment or surgical resection of MVNT lesions. Surgical resection is recommended when seizures are definitively related to MVNT and there is no therapeutic response to epileptic seizures and/or severe headaches [1,2]. The biopsy of the lesions makes the definitive diagnosis; however, this invasive procedure carries significant risks, given the location of most of these MVNT lesions.

## Conclusions

MVNT can be accurately diagnosed based on distinct MRI characteristics, often eliminating the need for biopsy or surgical intervention. In our patient, radiological stability was demonstrated between the baseline MRI and the 12-month follow-up MRI, accompanied by good seizure control. These observations underscore the benign, indolent nature of MVNT. Recognition of its distinct imaging characteristics is essential to prevent unnecessary invasive procedures, and annual MRI surveillance remains a safe and evidence-based management strategy.
